# “Screening at the door” - continuous rapid molecular screening for MRSA at emergency department is efficacious and effective

**DOI:** 10.1186/2047-2994-4-S1-P189

**Published:** 2015-06-16

**Authors:** BSP Ang, SY Tay, M Umayam, BF Poh, PU Krishnan

**Affiliations:** 1 Infectious Diseases, Tan Tock Seng Hospital, Singapore, Singapore; 2 Emergency, Tan Tock Seng Hospital, Singapore, Singapore; 3 Laboratory Medicine, Tan Tock Seng Hospital, Singapore, Singapore; 4 Infection Control, Tan Tock Seng Hospital, Singapore, Singapore

## Introduction

MRSA is highly prevalent in Singapore hospitals. MRSA bacteremia is being monitored by Ministry of Health (MOH) and will become a key performance indicator from 2015.

TTSH is a 1600 bedded hospital with the second busiest emergency department in the country. The majority of patients are in open, multi-bedded wards and shared facilities.

Many strategies including active surveillance for MRSA and cohorting of patients have been in place for several years. Patients are screened on admission, and positive patients transferred to cohort wards with substantial resource wastage as well as increased chances of transmission.

## Objectives

The objective of this study was to see if implementation of rapid molecular screening for MRSA before admission would improve cohorting, reduce post-admission transfers and MRSA infection rates.

## Methods

Estimates of time and effort spent on transferring of patients were made by observation and questioning of nursing and medical staff.

We implemented screening for MRSA at the Emergency department in 2012, using polymerase chain reaction (PCR) after setting up a satellite laboratory just next to the ED. Patients confirmed for admission had nasal swabs done by trained medical technicians who then did the PCR test. Results were ready within 2 -3 hrs and routed to Bed Management Unit who then assigned patients to appropriate wards.

## Results

Each patient transfer entailed an average of 155 min of nursing and housekeeping and 30 min of doctor’s time. After implementation of screening before bed assignment, there was reduction in transfers of patients to cohort wards from 8% to 2% of all admissions (OR 0.23; 95%CI 0.19, 0.29; P=0.0000).

Comparing 2014 to 2012, there was a 22% reduction in MRSA bacteremia rates (OR 0.78; 95% CI 0.53, 1.14; *P*=0.1853).

## Conclusion

Continuous rapid molecular screening for MRSA at Emergency Department is Efficacious and Effective in reducing transfers and MRSA bacteremia rates and has been sustainable.

## Disclosure of interest

None declared.

